# Improving quality of life with nutritional supplementation in Schizophrenia: A literature review

**DOI:** 10.1192/j.eurpsy.2022.1524

**Published:** 2022-09-01

**Authors:** N. Hassan, N. Dumlao, K. Tran, A. Zamiri

**Affiliations:** 1 Bronx Care Health System, Psychiatry, Bronx, United States of America; 2 BronxCare Health System, Psychiatry, Bronx, United States of America; 3 BronxCare Health System, Psychiatry, New York, United States of America

**Keywords:** “schizophrenia” “nutrition” “supplements”, “omega 3’s” “iron” “vitamin D” “vitamin C”, “sarcosine”, “D-serine” “sodium benzoate”

## Abstract

**Introduction:**

Schizophrenia is a chronic and severe mental health disorder, affecting 20 million people worldwide. Diet is a social determinant of health and is among one of the modifiable prognostic factors for schizophrenics. Previous research in nutritional psychiatry has shown that a balanced and healthy diet in this patient population has the potential to improve cognition, decrease positive and negative symptoms of the disease, and improve the overall metabolic profile.1,3

**Objectives:**

To understand the evidence on the role that nutritional supplements play in improving quality of life in Schizophrenia by improving cognitive symptoms and decrease mortality by decreasing chances of metabolic syndrome and CVD. Demonstrate how certain supplements can improve cognitive symptoms, and decrease positive and negative symptoms in schizophrenics

**Methods:**

PubMed was used to search for articles within the past 10 years

**Results:**

A total of 29 articles were initially generated, of which only 5 fit the search criteria. Each specific search produced more articles, and after carefully reading each, a total of 14 articles was determined to fit the criteria. All, but two articles included PANSS score assessment. The studies on vitamin D, cycloserine and omega 3’s produced conflicting

**Conclusions:**

Supplementation of vitamin D, Konjac powder, D-cycloserine, sarcosine, and omega 3’s have the potential to improve symptomatology and enhance the quality of life of schizophrenics. D-serine and sodium benzoate have not been shown to be effective adjunctive treatments in schizophrenia. Due to a limited number of studies for each, more research is indicated to truly determine the public health significance.

**Disclosure:**

No significant relationships.

